# Probiotic Enhancement of Antioxidant Capacity and Alterations of Gut Microbiota Composition in 6-Hydroxydopamin-Induced Parkinson’s Disease Rats

**DOI:** 10.3390/antiox10111823

**Published:** 2021-11-17

**Authors:** Shu-Ping Tsao, Bira Arumndari Nurrahma, Ravi Kumar, Chieh-Hsi Wu, Tu-Hsueh Yeh, Ching-Chi Chiu, Yen-Peng Lee, Yi-Chi Liao, Cheng-Hsieh Huang, Yao-Tsung Yeh, Hui-Yu Huang

**Affiliations:** 1 Ph.D. Program in Drug Discovery and Development Industry, College of Pharmacy, Taipei Medical University, Taipei 11031, Taiwan; d343105004@tmu.edu.tw; 2 Graduate Institute of Metabolism and Obesity Sciences, College of Nutrition, Taipei Medical University, Taipei 11031, Taiwan; ma48108006@tmu.edu.tw (B.A.N.); ravikumar@tmu.edu.tw (R.K.); lyp0913@tmu.edu.tw (Y.-P.L.); ma48109004@tmu.edu.tw (Y.-C.L.); 3 School of Pharmacy, Taipei Medical University, Taipei 11031, Taiwan; chhswu@tmu.edu.tw; 4 Department of Neurology, Taipei Medical University Hospital, Taipei 11031, Taiwan; do2739@tmu.edu.tw; 5 School of Medicine, Taipei Medical University, Taipei 11031, Taiwan; 6 Neuroscience Research Center, Chang Gung Memorial Hospital at Linkou, Taoyuan 33305, Taiwan; ccchei@mail.cgu.edu.tw; 7 Department of Nursing, Chang Gung University of Science and Technology, Taoyuan 33305, Taiwan; 8 Department of Medical Biotechnology and Laboratory Science, College of Medicine, Chang Gung University, Taoyuan 33305, Taiwan; 9 Aging and Disease Prevention Research Center, Fooyin University, Kaohsiung 83102, Taiwan; prevailingkimo@gmail.com (C.-H.H.); glycosamine@yahoo.com.tw (Y.-T.Y.); 10 Program in Environmental and Occupational Medicine, Kaohsiung Medical University, Kaohsiung 80708, Taiwan; 11 Biomedical Analysis Center, Fooyin University Hospital, Pingtung 92849, Taiwan; 12 Department of Medical Laboratory Sciences and Biotechnology, Fooyin University, Kaohsiung 83102, Taiwan

**Keywords:** probiotics, neuroprotection, fecal microbiota composition, antioxidant activities, Parkin-son’s disease

## Abstract

Oxidative stress plays a key role in the degeneration of dopaminergic neurons in Parkinson’s disease (PD), which may be aggravated by concomitant PD-associated gut dysbiosis. Probiotics and prebiotics are therapeutically relevant to these conditions due to their antioxidant, anti-inflammatory, and gut microbiome modulation properties. However, the mechanisms by which probiotic/prebiotic supplementation affects antioxidant capacity and the gut microbiome in PD remains poorly characterized. In this study, we assessed the effects of a *Lactobacillus salivarius* AP-32 probiotic, a prebiotic (dried AP-32 culture medium supernatant), and a probiotic/prebiotic cocktail in rats with unilateral 6-hydroxydopamine (6-OHDA)-induced PD. The neuroprotective effects and levels of oxidative stress were evaluated after eight weeks of daily supplementation. Fecal microbiota composition was analyzed by fecal 16S rRNA gene sequencing. The supplements were associated with direct increases in host antioxidant enzyme activities and short-chain fatty acid production, protected dopaminergic neurons, and improved motor functions. The supplements also altered the fecal microbiota composition, and some specifically enriched commensal taxa correlated positively with superoxide dismutase, glutathione peroxidase, and catalase activity, indicating supplementation also promotes antioxidant activity via an indirect pathway. Therefore, *L. salivarius* AP-32 supplementation enhanced the activity of host antioxidant enzymes via direct and indirect modes of action in rats with 6-OHDA-induced PD.

## 1. Introduction

Parkinson’s disease (PD), the most rapidly increasing neurodegenerative disease, mainly affects older individuals (~1% of >60-year-olds and 4% of >80-year-olds) [1,2]. The major clinical symptoms of PD are movement-associated motor dysfunctions, such as tremors, bradykinesia, gait disturbances, postural instability, and muscle rigidity [3]. These symptoms are generally treated with medications, such as levodopa, monoamine oxidase type B inhibitors, and either dopamine agonists or anti-cholinergics [4]. The clinical features of PD also extend to non-motor dysfunctions, such as cognitive impairment, psychosis, depression, anxiety, sleep disorders, and gastrointestinal (GI) issues [5]. The motor symptoms are related to progressive, selective loss of dopaminergic neurons in the substantia region of the brain caused by abnormal accumulation of intracellular α-synuclein (α-syn) as well as the formation of Lewy bodies that induce aberrant basal ganglia function [6]. Risk factors, such as gene mutations (α-syn, parkin), brain injury, stress, and pesticide exposure, are also linked to PD [7]. Although the origin of PD is not fully understood, recent studies showed that the non-motor symptoms—particularly GI dysfunction—manifest before the premotor phase of PD and may contribute to PD pathogenesis via the gut–brain axis [8,9].

The GI tract hosts and maintains a symbiotic relationship with a diverse community of microbes. The composition of the gut microbiota is affected by various factors, such as lifestyle, diet, drug treatments, geolocation, GI surgery, age, gender, host genotype, and delivery mode (cesarean vs. vaginal) [10]. A healthy and stable intestinal microbiota composition plays multi-faceted roles in the maintenance of immune homeostasis [11] and metabolism [12], gut barrier integrity [13] and function, and the gut–brain interaction [14]. Recent studies indicate that alterations to the gut microbiome may potentially play roles in mental disorders, such as autism [15], schizophrenia [16], anxiety, and depression [17]. Other recent studies reported phenotypic correlations between PD pathogenesis and the gut microbiota [18] and microbe-derived metabolites, specifically short-chain fatty acids (SCFAs) [19]. SCFAs are important components of the interplay between diet, gut microbiota, and downstream sensitization of inflammatory cascades [20]. Compared to healthy individuals, patients with PD frequently exhibit gut microbiota dysbiosis, which is characterized by a significant decrease in the abundance of *Prevotellaceae* in fecal samples [21]. Moreover, the abundance of Enterobacteriaceae directly correlates with the severity of gait difficulties and posture instability in PD [22]. Hence, an improved understanding of the linkages between the gut microbiome and the development and progression of PD could possibly enable earlier diagnosis and improved treatment of PD.

The growing evidence of a bidirectional relationship between the gut and the brain suggests probiotics could potentially be used to improve gut microbiota health and stability and to prevent or delay neurodegenerative diseases. The anti-inflammatory and antioxidant properties of probiotics and their ability to modulate the composition of the gut microbiota may contribute to the prevention of neuronal damage. In our previous study, we showed that probiotic/prebiotic supplementation increased glycolysis and mitochondrial activity and potentially increased energy metabolism in the muscle and brain in a rat model of PD. Consequently, these effects may prevent muscle atrophy and loss of dopaminergic neurons. The supplementation also modulated the production of SCFAs and increased the activity of antioxidative enzymes. These changes could protect mitochondria against damage induced by reactive oxygen species (ROS) [23]. Several studies have documented the immunomodulatory function of probiotics [24,25,26]. Supplementation of probiotics has been shown to reduce oxidative stress by limiting lipid peroxidation and increasing the native levels of superoxide dismutase (SOD) in a mouse model of stroke [27]. Additionally, probiotics could restructure the composition of the microbiota in the GI tract, which may enhance host anti-inflammatory and antioxidant capacity. Probiotics have been reported to decrease the numbers of pathogenic bacteria and increase anti-inflammatory-related bacteria and thus result in decreased levels of pro-inflammatory cytokines [28]. Restructuring of the gut microbiota composition with probiotics was also shown to modulate production of the SCFAs, indole-3-propionic acid, and the phenol ferulic acid, among other metabolites, which could inhibit oxidative stress by balancing excessive production of ROS [29].

*Lactobacillus* and *Bifidobacterium*, the most commonly used probiotics, can generate the SCFAs acetic acid, lactic acid, and propionic acid. These probiotics can also lower the pH of the intestine and suppress the proliferation of pathogenic bacteria [30,31]. Probiotic treatment alters the composition the microbiota along the digestive tract. For example, twelve days of treatment with *Lactobacillus* GG enhanced the dominance of the facultative anaerobe *Lactobacillus rhamnosus GG* as well as other anaerobic and aerobic bacteria in fecal samples [32]. However, not all probiotic strains affect the composition or diversity of the gut microbiota. Supplementation with *Bifidobacterium* BB-12, W23, W52, W58, or Bi-07 or *Lactobacillus rhamnosus* DR20, LGG, NCFM, or LA-5 for six weeks to six months enhanced the abundance of *Bifidobacterium* or *Lactobacillus* strains in the luminal microbiota but did not change the gut microbiota composition or diversity [33,34,35]. Several reports have validated that manipulation of the gut microbiota using various probiotic strains may affect host metabolism, promote gut barrier reinforcement, and reduce inflammation [36,37].

We hypothesized that probiotic supplementation may confer a neuroprotective effect and attenuate motor deficit in rats with 6-hydroxydopamine (6-OHDA)-induced PD. Furthermore, only a few studies have specifically focused on the mechanisms of action of probiotics on the gut microbiota dysbiosis in PD. Therefore, through this study, we aimed to examine the neuroprotective effects of supplementation with the probiotic *Lactobacillus salivarius* AP-32 and prebiotic (medium residue obtained from AP-32 culture) in a rat model of PD in order to gain more insight into the relationship between probiotics and the gut microbiota dysbiosis in PD. We present new information on the characteristics of the fecal microbiota in rats with 6-OHDA-induced PD. Our study shows that probiotic/prebiotic supplementation promotes neuroprotection of the dopaminergic neurons and ameliorates the deterioration in motor function in a rat model of PD by suppressing PD-associated oxidative stress. We show these effects are associated with direct and indirect upregulation of antioxidant pathways and thus improved antioxidant capacity. The direct pathway involves induction of increased antioxidant enzyme activities and SCFA production by the supplements, while the indirect pathway involves supplementation-induced reshaping of the gut microbiota composition to facilitate microbial metabolite production and antioxidant activity. Thus, this study provides new evidence on the linkages between probiotic/prebiotic supplementation and antioxidant optimization via alterations of the fecal microbiota in a PD-like model.

## 2. Materials and Methods

### 2.1. Supplements

The probiotic and potential prebiotic were supplied by Bioflag Biotech Co., Ltd. (Tainan, Taiwan). *Lactobacillus salivarius* subsp. *salicinius* AP-32 (10^11^ CFU/g) probiotic was isolated by Bioflag Biotech Co., Ltd., from a healthy human gut and deposited in collections at the Food Industry Research and Development Institute, Hsinchu City, Taiwan, and Wuhan University, Hubei China. The culture medium residue (MR) prebiotic consists of spray-dried, bacteria-free supernatant obtained from fermented *Lactobacillus salivarius* subsp. *salicinius* AP-32 culture broth. MR contains numerous nutrients, including macronutrients, amino acids, and SCFAs [38]. Synbiotic 1XMR (Bioflag Biotech Co., Ltd.) is a combination of the probiotic AP-32 and potential prebiotic MR. All supplements were dissolved in distilled water (1 mg/mL) before oral gavage.

### 2.2. Animals and Study Design

Sprague–Dawley rats (male, eight-weeks-old, weight 290 ± 10 g) were obtained from Bio-LASCO Taiwan Co., Ltd., Taipei, Taiwan. The animals were housed (one animal per cage) in a controlled environment under a 12-h dark and 12-h light cycle, temperature range of 22–24 °C, and relative humidity of 40–60% at the Laboratory Animal Center of Taipei Medical University, Taipei, Taiwan. Water and food were provided ad libitum.

Rats were randomly allocated to ND (non-diseased, *n* = 5) and PD (*n* = 25) groups. After the apomorphine test (see below) (6 weeks after induction of 6-OHDA lesions (see below)), the rats with PD were randomly allocated to the following groups: PD (untreated PD, *n* = 5), LD (PD treated with 8 mg of L-DOPA, *n* = 5), 1X (PD supplemented with 1.03 × 10^9^ CFU/kg BW of probiotic, *n* = 5), MR (PD supplemented with 62 mg/kg BW of MR, *n* = 5), and 1XMR (PD supplemented with a combination of 1X and MR, *n* = 5). All supplements were administered via oral gavage once a day for 8 weeks. The inoculum volume of probiotic given to each rat was 0.3–0.6 mL, according to the rat’s BW. This volume was equal to 0.3 × 10^9^ CFU to 0.6 × 10^9^ CFU for 300–600 g BW of rat. To compare the probiotic and prebiotic effects with the effects of commonly used medication (L-DOPA) for PD, rats in the LD group were intraperitoneally injected with 8 mg/kg BW of L-DOPA along with 15 mg/kg BW benserazide (Sigma-Aldrich, Darmstadt, Germany) (dissolved in 0.9% NaCl) three times per week for 8 weeks (total 24 times), modified from [39]. Benserazide is a dopamine decarboxylase inhibitor that was used to prevent the conversion of L-DOPA into dopamine in the bloodstream. Prior to injection in the rats, the L-DOPA solution was filter sterilized using sterile syringe filter (Acrodisc^®^, Pall Corporation, San Juan, NC, USA) in a laminar flow hood. The ND and PD groups received water via oral gavage as a placebo. The bodyweights (BW) of the rats were assessed weekly, and water and food consumption were recorded daily.

After the 8-week supplementation period, motor function was assessed by behavioral tests (see below). On the final day of supplementation, fecal samples were collected to evaluate the composition of the fecal microbiota and microbial metabolites, then the rats were humanely sacrificed by anesthetization with anesthetic agents followed by intracardiac perfusion. Anesthetic agents were tiletamine-zolazepam (20–40 mg/kg BW; Zoletil^®^, Virbac, New Zealand Ltd., Hamilton, New Zealand) and xylazine (5 to 10 mg/kg BW; Rompun^®^, Bayer Health Care, Leverkusen, Germany). Intracardiac perfusion was performed using 0.9% NaCl followed by 4% paraformaldehyde (200 mL).

The in-vivo experiments were approved in advance by the Taipei Medical University Institutional Animal Care and Use Committee (IACUC) and performed in compliance with the Taiwan code of animal use and care for scientific objectives (approval no. LAC-2020-0183).

### 2.3. Unilateral 6-OHDA Lesions

Unilateral 6-OHDA lesions were induced by stereotaxic surgery using a modified version of a protocol from a previous study [38]. Briefly, 20–40 mg/kg BW of tiletamine with zolazepam (Zoletil^®^, Virbac, New Zealand Ltd., Hamilton, New Zealand) and 5 to 10 mg/kg BW of xylazine (Rompun^®^, Bayer Health Care, Leverkusen, Germany) were used to anesthetize the rats before surgery. A stereotaxic apparatus for rats (David Kopf Instruments, Tujunga, CA, USA) was used to restrain the rats. A 1-mm burr hole was created, using a micro motor handpiece (Saeshin Production Co., Ltd., Daegu, Korea), on the coordinates of the right medial forebrain bundle [38]. Freshly prepared 3 μg/μL 6-OHDA solution (6-OHDA in 0.9% NaCl containing 0.02% ascorbic acid (*w*/*v*); Sigma-Aldrich, Darmstadt, Germany) was injected with a 10-μL Hamilton syringe using an infusion pump at 0.2 μL/min. The syringe was left in place for 5 min after injection, then gently retracted. The same procedure was performed on NC rats, using 3 μL of 0.9% NaCl containing 0.02% ascorbic acid (*w*/*v*). A heating pad was used to keep the animals warm during the entire surgical and recovery procedures. All injected solutions were filter sterilized using sterile syringe filter (Acrodisc^®^, Pall Corporation, San Juan, NC, USA) in the laminar flow hood before injecting into the brain. Six weeks after 6-OHDA injection, apomorphine-rotation tests (see below) were performed to confirm the induction of PD. Rats with contralateral rotation of the head away from the injection site over more than 100 rotations in 1 h were included as PD rats.

### 2.4. Evaluation of Neuroprotective Effects

#### 2.4.1. Immunohistochemical Staining and Image Analysis

Dopaminergic neuronal populations in the striatum and substantia nigra were evaluated via tyrosine hydroxylase (TH) immunohistochemical staining. After the rats were humanely sacrificed using anesthetic agents and intracardially perfused, their brains were rapidly extracted from their skull and incubated in 4% paraformaldehyde for 24 h at 4 °C. The forebrain (containing striatum) and midbrain (containing substantia nigra) tissues were embedded in paraffin blocks. Coronal slices were prepared in the coordinates of the striatum and substantia nigra. The coronal brain sections were obtained using a microtome at 5-µm thickness. Slides were deparaffinized and hydration, then incubated in 0.3% hydrogen peroxide solution for 10 min under dim light conditions, then in PBS for 1 h at room temperature (RT), followed by primary antibody rabbit polyclonal anti-TH (Elabscience, Houston, TX, USA, Cat. No. E-AB-70077; diluted 1:300) for 24 h at 4 °C, then washed, followed by incubation with goat anti-rabbit IgGHRP (Sigma, B7401; diluted 1:100) for 2 h at room temperature. Primary and secondary antibodies were diluted in PBS containing 0.4% Triton X-100. The chromogen 3,3′-diamino-benzidine (DAB Substrate Kit for Peroxidase, Vector, Burlingame, CA, USA) was used to reveal the immunocomplexes, then the sections were washed three times with 0.1 M PBS for 15 min each. Slides were then dehydrated by brief immersion in distilled water (three times), in 96% ethanol (three times), 99% ethanol (one time, 2 min), and in histoclear (two times, 2 min each), followed by mounting with cover slips using Permount™ mounting media (Fisher Scientific) according to the manufacturer’s instructions. Images were captured using Motic EasyScan (Motic, Hong Kong). ImageJ was used to quantify the TH-positive staining intensity. The final quantification of TH-positive intensity is presented as a percentage of the non-lesioned side.

#### 2.4.2. Behavioral Tests

Motor function was evaluated using a variety of behavioral tests. The apomorphine-induced rotation test (rotation of the head away from the injection site over more than 100 rotations in 1 h) assessed the severity of dopamine deprivation in the 6-OHDA-lesioned part of the brain by monitoring contralateral rotations induced by apomorphine. Apomorphine was administered subcutaneously (0.5 mg apomorphine/kg BW in 0.9% NaCl containing 1% ascorbic acid; Sigma-Aldrich). The apomorphine-rotation test was conducted 6 weeks after injection of 6-OHDA to confirm establishment of the PD model and after 8 weeks of supplementation to evaluate the effects of the treatments. The rotarod test was conducted after 8 weeks of supplementation to evaluate locomotor function. The rotarod test was performed at 10 rpm using a 7650 Rotarod (Ugo Basile, Collegeville, PA, USA) for a maximum duration of 2 min. The time spent on the rod was recorded.

### 2.5. Evaluation of Oxidative Stress and Inflammation Markers in Serum

#### 2.5.1. ROS Assay

Oxidative stress status was investigated by assessing the levels of ROS and activity of antioxidant enzymes in serum after 8 weeks of supplementation. ROS were quantified using the DCF ROS/RNS assay kit (Abcam, Cambridge, UK; Cat. No. Ab238535) in accordance with the manufacturer’s protocol. Briefly, 50 µL of samples or hydrogen peroxide standards were added into a black 96-well plate. Catalyst (50 µL) was added into each well, incubated for 5 min at RT, then DCFH solution (100 µL) was added and incubated for 30 min in the dark at RT. Fluorescence was read at 480 nm/530 nm (excitation/emission).

#### 2.5.2. Quantification of Antioxidant Enzyme Activity

The activity of superoxide dismutase (SOD), glutathione peroxidase (GPx), and catalase (CAT) in serum were quantified using commercial colorimetric assays (BioVision, Milpitas, CA, USA) in accordance with the manufacturer’s protocol.

#### 2.5.3. Quantification of Inflammatory Cytokines

Inflammation was evaluated after 8 weeks of supplementation by analyzing the levels of the inflammatory cytokine tumor necrosis factor alpha (TNF-α) in serum using a commercial Sandwich ELISA kit pre-coated with antibody (Elabscience, Houston, TX, USA; Cat. No. E-EL-R0019) according to the manufacturer’s instructions. Briefly, samples or standards (100 µL) were added into the 96-well plate coated with anti-rat TNF-α, then Biotinylated Detection Ab (100 µL) was added into every well. The plate was sealed, incubated for 1 h at 37 °C, each well was rinsed three times using wash buffer, incubated with HRP Conjugate (100 µL) for 30 min at 37 °C, washed five times with wash buffer, and then incubated with Substrate Reagent (90 µL) at 37 °C while protected from light. After 15 min, Stop Solution (50 µL) was added to end the reaction. The optical density reading was taken at 450 nm.

### 2.6. Evaluation of Microbiota Composition and Microbial Metabolites

#### 2.6.1. Bacterial Genomic DNA Isolation

The day after the 8-week-supplementation period ended, fresh fecal samples (200 mg) were collected from individual rat by abdominal massage to expedite the process of defecation. Total genomic DNA was extracted using the QIAamp DNA Stool Mini kit (Qiagen, Hilden, Germany) following the protocol provided by the manufacturer. DNA concentrations were measured using a NanoDrop2000 (Thermo Scientific, Waltham, MA, USA). The fecal samples were stored at −80 °C.

#### 2.6.2. 16S rRNA Amplicon Sequencing and Data Analysis

The Illumina 16S Metagenomic Sequencing Library Preparation protocol was employed to generate libraries from the V3-V4 region of the 16S rRNA gene by PCR with the primers 341 F (5′-CCTAYGGGRBGCASCAG-3′) and 806R (5′-GGACTACNNGGGTATCTAAT-3′) [40]. The amplicons were paired-end sequenced (PE 2 × 250) on an Illumina HiSeq 2000 platform, following the manufacturer’s protocol. Quantitative Insights into Microbial Ecology (QIIME) was used to analyze the data [41]. After the quality filtering, the paired forward and reverse-reads were merged. The UPARSE algorithm was applied to align identical sequences and determine representative operational taxonomic unit (OTU) sequences. Sequences that were ≥97% identical were clustered to the same OTU. Chimeric sequences were removed using ChimeraSlayer. The SILVA v132 database was used to annotate the OTUs. Alpha diversity analysis (Shannon index) was performed using QIIME. Beta diversity was analyzed using weighted principal coordinate analysis (PCoA) and partial least squares discriminant analysis (PLS-DA) with QIIME. The online Galaxy workflow framework was utilized to determine the linear discriminant analysis (LDA) effect size (LefSe).

#### 2.6.3. High-Performance Liquid Chromatography (HPLC)

Fecal pellets were collected from individual rat by abdominal massage after the supplementation period ended. The SCFA contents of the fecal samples were determined via HPLC using a previously described protocol [23]; we analyzed the concentrations of acetic, propionic, butyric, isobutyric, valeric, and isovaleric acid. Fecal SCFAs were extracted from each rat’s feces by mixing 200 mg of the feces with 5 mL of 70% ethanol. The mixtures were centrifuged for 10 min at 3000× *g* at 20 °C. The mixtures were centrifuged for 10 min at 3000× *g* at 20 °C, the supernatants were collected, and 1-EDC-HCl, pyridine, 2-ethylbutyric acid, and 2-NPH-HCl were added to the supernatants. The mixtures were incubated at 60 °C; after 20 min, 0.2 mL of potassium hydroxide was added, and the mixtures were incubated at 60 °C for another 20 min. After incubation, the mixtures were placed in room temperature until cooled down. After that, aqueous phosphoric acid and ether (≥99%) were added, and the mixtures were shaken for 3 min and centrifuged (3000× *g*, 10 min, 20 °C). The ether layer was collected in a new tube. Water was added to the ether layer, shaken for 3 min, and centrifuged (3000× *g*, 10 min, 20 °C). The ether layer was collected, and ether was eliminated by streaming nitrogen gas across the surface to generate fatty acid hydrazides. Next, the generated fatty acid hydrazides were dissolved in 0.1 mL of methanol and subjected to HPLC. An aliquot of 30 μL was separated in a YMC-Pack FA 250 × 6 nm ID column (YMC, Kyoto, Japan) with temperature of 50 °C and at 1.1 mL/minute flow rate. The concentrations of SCFAs were measured at 400 nm.

### 2.7. Statistical Analyses

Data are presented as the mean ± standard error of the mean (SEM) and were analyzed using one-way ANOVA with Tukey HSD post-hoc tests. The correlations between bacteria and motor functions and between bacteria and SCFAs were analyzed using Pearson’s correlation analysis. A *p*-value < 0.05 was considered statistically significant.

## 3. Results

### 3.1. Long-Term Probiotic and Prebiotic Supplementation Ameliorated General Motor Symptoms and Confered Neuroprotective Effects in Rats with 6-OHDA-Induced PD

**Figure 1:**
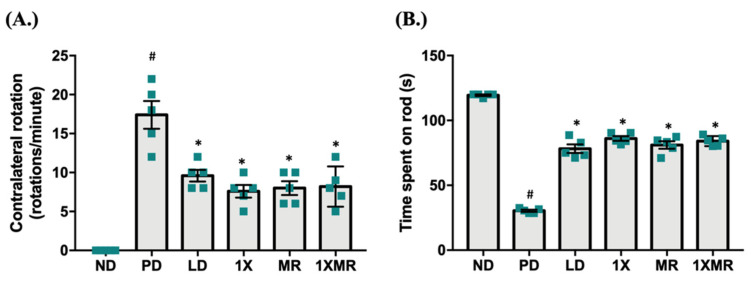
Evaluation of motor function in the ND, PD, LD, 1X, MR, and 1XMR groups after 8 weeks of treatment. (**A**) Contralateral rotations during the apomorphine-induced rotation test. (**B**) Endurance performance in the rotarod test. Data are expressed as mean ± SEM (*n* = 5 rats/group). Significant differences (bars) are expressed as *p* < 0.05. One-way ANOVA with Tukey’s post hoc test; # *p* < 0.05 for comparison of the PD group with the ND group; * *p* < 0.05 for comparison of LD, 1X, MR, and 1XMR groups with the PD group. ND, non-diseased; PD, untreated PD; LD, PD treated with 8 mg of L-DOPA; 1X, PD supplemented with the probiotic; MR, PD supplemented with MR as prebiotic; 1XMR, PD supplemented with a combination of 1X and MR.

To evaluate the effects of the treatments on development of the characteristic motor symptoms of PD, the apomorphine test was performed at eight weeks after 6-OHDA injection. Additionally, balance skills and motor coordination were assessed using the rotarod test (Figure 1). In the apomorphine test, ND rats did not exhibit contralateral rotation, whereas the rats in PD group exhibited a high frequency of contralateral rotations (Figure 1A). The rats in the LD, 1X, MR, and 1XMR treatment groups exhibited significantly lower frequencies of contralateral rotation than the untreated PD group. In the rotarod test, the PD rats showed a significantly lower (*p* < 0.05) endurance performance than the ND rats (Figure 1B). Compared to the untreated PD rats, the rats in the LD, 1X, MR, and 1XMR treatment groups showed significantly higher (*p* < 0.05) endurance performances. The treated PD rats exhibited reduced (*p* < 0.05) severities of motor dysfunction and improved (*p* < 0.05) endurance performances compared to the untreated PD rats. These results indicated that probiotic/prebiotic supplementation alleviated motor dysfunction in the 6-OHDA-induced rat model of PD.

**Figure 2:**
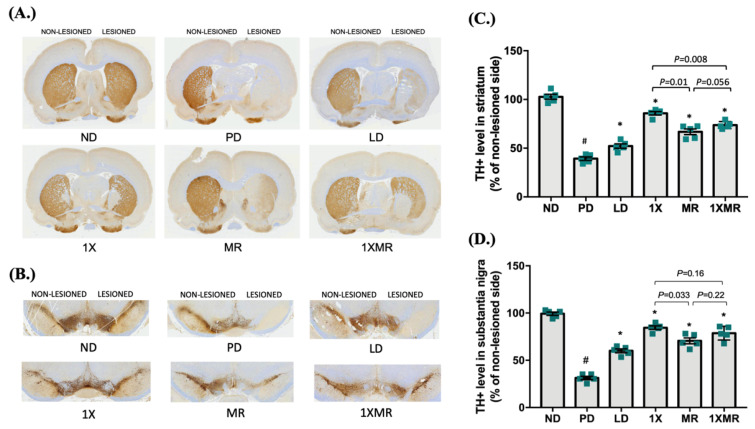
Evaluation of dopaminergic neurons in the striatum and substantia nigra pars compacta (SNpc) after 8 weeks of treatment. (**A**) Immunohistochemical staining for tyrosine hydroxylase (TH) was performed to identify dopaminergic neurons in the striatum. (**B**) Immunohistochemical staining of TH in the SNpc region. (**C**) Quantification of TH-positive (TH+) cells in the striatum. (**D**) Quantification of TH-positive cells in the SNpc region. TH staining intensity is normalized to the non-lesioned side and expressed as mean ± SEM (*n* = 5 rats/group). Significant differences (bars) are expressed as *p* < 0.05. One-way ANOVA with Tukey’s post hoc test; # *p* < 0.05 for comparison of the PD group with the ND group; * *p* < 0.05 for comparison of LD, 1X, MR, and 1XMR groups with the PD group. ND, non-diseased; PD, untreated PD; LD, PD treated with 8 mg of L-DOPA; 1X, PD supplemented with the probiotic; MR, PD supplemented with MR as prebiotic; 1XMR, PD supplemented with a combination of 1X and MR.

To validate that the improvements in motor function in the treated groups were related to attenuation of the effects of 6-OHDA on dopaminergic neurons, we next investigated tyrosine hydroxylase (TH) expression in the striatum and substantia nigra regions of each group via immunohistochemical staining. (Figure 2A,B, respectively). In each section, TH staining intensity in the lesioned side was expressed relative to that of the non-lesioned side. The ND group retained nearly 100% of the dopaminergic neurons on the lesioned side (Figure 2C), whereas the PD group showed a significant (*p* < 0.05) reduction in the TH-positive signal in the lesioned striatum region. However, all PD treatment groups showed significantly higher (*p* < 0.05) TH-positive signals in the striatum than the PD group; the 1X group showed the highest (*p* < 0.05) TH-positive level on the lesioned side, followed by the 1XMR and MR groups. Similar trends in the TH-positive levels were observed in the lesioned substantia nigra region (Figure 2D). Induction of sham lesions with saline in the ND group did not affect the population or function of dopaminergic neurons on the sham-lesioned side. However, induction of 6-OHDA lesions resulted in near total loss of dopaminergic neurons in the striatum and substantia nigra regions in the PD group. However, the LD, 1X, MR, and 1XMR treatments reduced the severity of the effects of 6-OHDA and mitigated the extent of dopaminergic neuronal loss. These results clearly indicate that long-term probiotic/prebiotic supplementation provided neuroprotective effects in the rat model of PD.

### 3.2. Probiotic and Prebiotic Supplementation Reduced Inflammation, Promoted Antioxidant Activity, and Increased SCFA Production

To investigate the link between the observed neuroprotective effects and oxidative stress, we next analyzed the levels of ROS, expression of an inflammatory marker, and the activity of antioxidant enzymes in serum (Table 1). Compared to the ND group, the PD group had significantly higher (*p* < 0.05) serum levels of ROS and TNF-α and lower (*p* < 0.05) serum SOD, GPX, and catalase activity. Serum ROS and catalase activity were similar (*p* > 0.05) in the LD group and PD group; however, serum TNF-α was significantly lower (*p* < 0.05), and SOD and GPX activity were significantly elevated (*p* < 0.05) in the LD group. In contrast, the 1X, MR, and 1XMR groups exhibited significantly lower (*p* < 0.05) serum ROS and TNF-α levels and significantly elevated (*p* < 0.05) SOD and GPX activity. The 1X and 1XMR groups both showed elevated (*p* < 0.05) catalase activity compared to the PD group though the MR group did not (*p* > 0.05). These results showed that supplementation with 1X, MR, and 1XMR probiotics for eight weeks reduced oxidative stress by increasing antioxidant activity at the systemic level. Taken together, these results strongly indicate that 1X, MR, and 1XMR supplementation not only prevented the development of systemic oxidative stress, but also attenuated the increase in the levels of circulating inflammatory markers in the rat model of PD.

**Figure 3:**
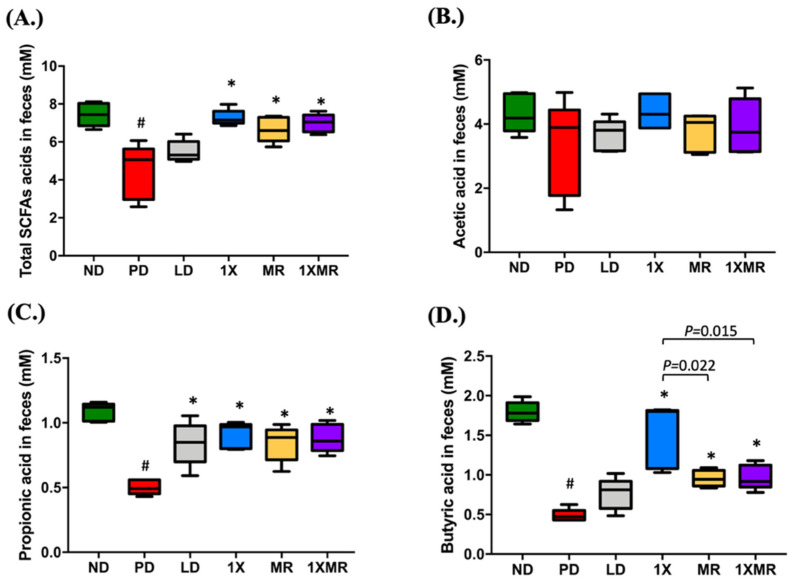
Effects of 8 weeks of supplementation with 1X, MR, and 1XMR on the fecal concentrations of (**A**) short-chain fatty acids, (**B**) acetic acid, (**C**) propionic acid, and (**D**) butyric acid. Values are reported as mean ± SEM for 5 rats per group. Significant differences (bars) are expressed as *p* < 0.05. One-way ANOVA with Tukey’s post hoc test; # *p* < 0.05 for comparison of the PD group with the ND group; * *p* < 0.05 for comparison of LD, 1X, MR, and 1XMR groups with the PD group. ND, non-diseased; PD, untreated PD; LD, PD treated with 8 mg of L-DOPA; 1X, PD supplemented with the probiotic; MR, PD supplemented with MR as prebiotic; 1XMR, PD supplemented with a combination of 1X and MR.

We further investigated the potential role of fecal microbial metabolites in modulation of anti-inflammatory and antioxidant activities by analyzing the fecal SCFA concentrations. Analysis of fecal samples revealed marked differences in the SCFA concentrations between groups. The total SCFA concentrations, representing the total for acetic, propionic, butyric, isobutyric, valeric, and isovaleric acids, are shown in Figure 3A. Compared to the ND group, the PD group had a significantly lower (*p* < 0.05) total SCFA concentration. The LD group had a similar (*p* > 0.05) total SCFA concentration as the PD group; however, the 1X, MR, and 1XMR groups had significantly higher (*p* < 0.05) total SCFA concentrations than the PD group. Analysis of the individual SCFAs further clarified the contribution of the constituent SCFAs. As shown in Figure 3B, there was no significant difference (*p* > 0.05) in the concentration of acetic acid between groups. The concentration of propionic acid was significantly lower (*p* < 0.05) in the PD group compared to the ND group (Figure 3C). Moreover, all treatment groups had higher (*p* < 0.05) propionic acid concentrations than the PD group. The butyric acid concentrations followed a similar trend as total SCFAs (Figure 3D). The PD group had a lower (*p* < 0.05) butyric acid concentration than the ND group though there was no difference (*p* > 0.05) between the LD and PD groups. Interestingly, the 1X group exhibited a very significantly higher concentration of butyric acid, even higher than that of the MR group (*p* = 0.022) and 1XMR group (*p* = 0.015). Therefore, the increase in total SCFAs in the probiotic/prebiotic treatment group was largely due to increased concentrations of propionic acid and butyric acid. Although LD treatment increased propionic acid production, the 1X, MR, and 1XMR treatments increased the production of both propionic acid and butyric acid. Notably, the production of beneficial butyric acid was more significantly enhanced by the 1X treatment than the other treatments. This result suggests supplementation of probiotic alone may have the most beneficial effects on the production of SCFAs in the feces.

### 3.3. Long-Term Probiotic and Prebiotic Supplementation Alter 6-OHDA-Induced Gut Dysbiosis by Remodeling the Composition of the Fecal Microbiota

**Figure 4:**
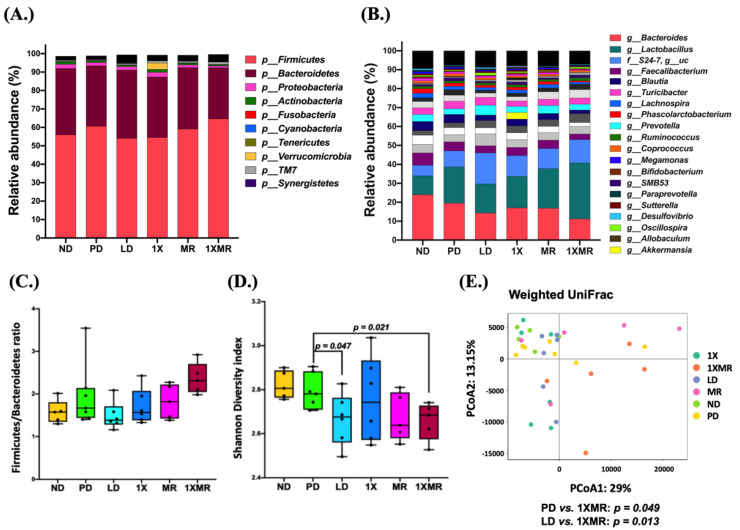
Comparison of bacterial relative abundance and bacterial diversity between groups (n = 5) after 8 weeks of treatment. (**A**) Distribution of the relative abundance of fecal microbiota at the phylum level. Only the top ten most abundant bacteria across all groups are displayed. (**B**) Relative distribution of the abundance of fecal microbiota at the genus level. Only the top 20 most abundant bacteria across all groups are displayed. (**C**) Ratio of *Firmicutes* to *Bacteroidetes* in the different groups. (**D**) Analysis of α-diversity based on the Shannon index. (**E**) Analysis of β-diversity using PCoA plots based on the weighted UniFrac metrics of fecal microbiota. ND, non-diseased; PD, untreated PD; LD, PD treated with 8 mg of L-DOPA; 1X, PD supplemented with the probiotic; MR, PD supplemented with MR as prebiotic; 1XMR, PD supplemented with a combination of 1X and MR.

We investigated the effects of the various treatments on the microbial composition of the fecal samples to gain insight into the possible linkages between the fecal microbiota composition and the observed therapeutic effects. After the eight-week-treatments, *Firmicutes* (57–68%), *Bacteroidetes* (29–39%), *Proteobacteria* (1–3%), *Actinobacteria* (~1%), *Saccharibacteria* (TM7) (~1%), and *Verrucomicrobia* (~1%) were the top six phyla across all groups. Inter-group variability in the relative abundance of the top ten phyla is shown in Figure 4A. The relative abundance of *Firmicutes* was increased, while *Bacteroidetes* decreased in the PD group compared to the ND group. Compared to the PD group, all treatment groups showed unique variability. Specifically, the 1X group showed a stark increase in *Verrucomicrobia*. Although there were noticeable differences in the relative abundance of *Firmicutes* and *Bacteroidetes* between groups, the *Firmicutes* to *Bacteroidetes* ratio did not vary greatly between the groups (Figure 4C).

Moreover, inter-group variability was also observed at the genus level. The relative abundance of the top 20 genera in the different groups are shown in Figure 4B. Compared to the ND group, the relative abundance of the major genera *Bacteroides* was decreased, and *Lactobacillus* was increased in the PD group. Compared to the PD group, all treatment groups had a higher abundance of *Lactobacillus* and lower abundance of *Bacteroidetes*. Specifically, the 1X group showed a remarkably increased abundance of *Akkermansia*.

Furthermore, changes in 𝛼-diversity based on the Shannon index were also observed between the groups (Figure 4D). Although the PD group did not show any change in 𝛼-diversity compared to the ND group, the treatments did change the microbiota diversity. Only the LD (*p* = 0.047) and 1XMR (*p* = 0.021) treatments led to lower 𝛼-diversity than the PD group. Analysis of the structure of the microbial communities based on PCoA plots (Figure 4E) for 𝛽-diversity showed significant differences between the PD and 1XMR groups (*p* = 0.049) and the LD and 1XMR groups (*p* = 0.013).

**Figure 5:**
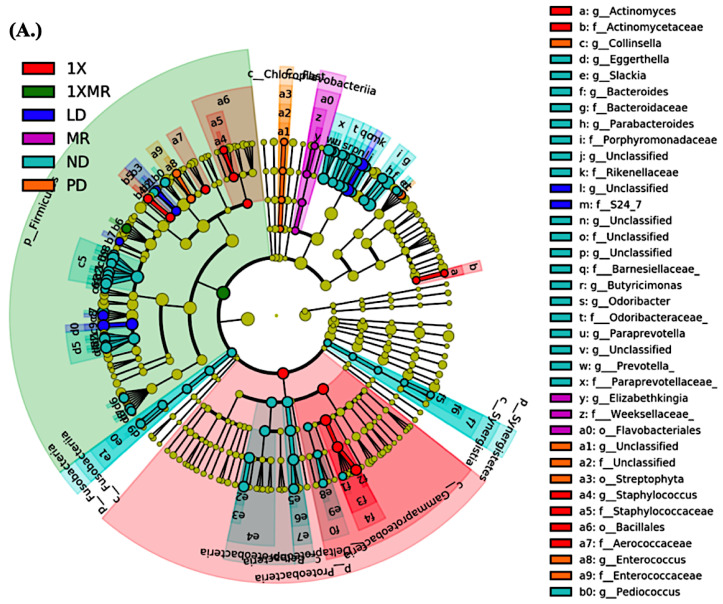

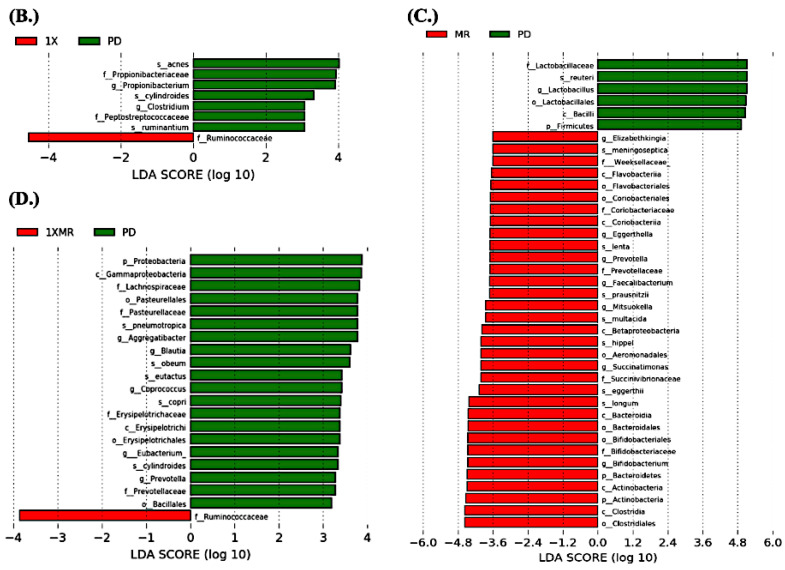
Comparison of the fecal microbiome between groups, highlighting the enriched taxa after 8 weeks of treatment. Linear discriminant analysis effect size (LEfSe) along with the identity of fecal bacterial markers that discriminated between the ND, PD, LD, 1X, MR, and 1XMR groups. (**A**) Linear discriminant analysis (LDA) scores for differentially abundant taxa between the ND, PD, LD, 1X, MR, and 1XMR groups are shown on the right. (**B**) Linear discriminant analysis (LDA) scores for differentially abundant taxa between the PD and 1X groups. (**C**) Linear discriminant analysis (LDA) scores for differentially abundant taxa between the PD and MR groups. (**D**) Linear discriminant analysis (LDA) scores for differentially abundant taxa between the PD and 1XMR groups. The input criteria for the LEfSe analysis included a LDA score > 3 (log 10) and an alpha value of 0.01 for the factorial Kruskal–Wallis test/pairwise Wilcoxon test. ND, non-diseased; PD, untreated PD; LD, PD treated with 8 mg of L-DOPA; 1X, PD supplemented with the probiotic; MR, PD supplemented with MR as prebiotic; 1XMR, PD supplemented with a combination of 1X and MR.

Linear discriminant analysis effect size (LEfSe) was conducted to identify the effects of the probiotic/prebiotic on the richness and patterns of abundance of specific taxa within each group. LEfSe analysis (LDA scores >3.0) comparing all groups is shown in Figure 5A, and it revealed that the relative abundance of eight taxa was different between the 1X supplemented and PD groups: *g_Propionibacterium*, *g_Clostridium*, *s_Cylindriodes*, and *s_ruminantium* were significantly increased in the PD group, while only f_Ruminococcaceae was increased in the 1X group (Figure 5B). Thirty-nine taxa were altered between the MR and PD groups, with the PD group showing a significant increase (*p* < 0.05) in *g_Lactobacillus* (Figure 5C). The MR group showed increases in *g_Elizabethkingia*, *g_Eggerthella*, *g_Prevotella*, *g_Faecalibacterium*, *g_Mitsuokella*, *g_Succinatimonas*, and *g_Bifidobacterium* compared to the PD group. Variability in 21 taxa was observed between the 1XMR and PD groups: *g_Aggregatibacter*, *g_Balutia*, *g_Coprococcus*, *g_Eubacterium*, and *g_Prevotella* were enriched in the PD group (Figure 5D). The 1XMR group only showed an increase in *f_Ruminococcaceae* compared to the PD group. These results indicate that the three supplements differently reshaped the fecal microbiome composition in the rat model of PD. Although 1X and 1XMR selectively enriched *f_Ruminococcaceae*, the MR treatment generally resulted in enrichment of multiple taxa.

**Figure 6:**
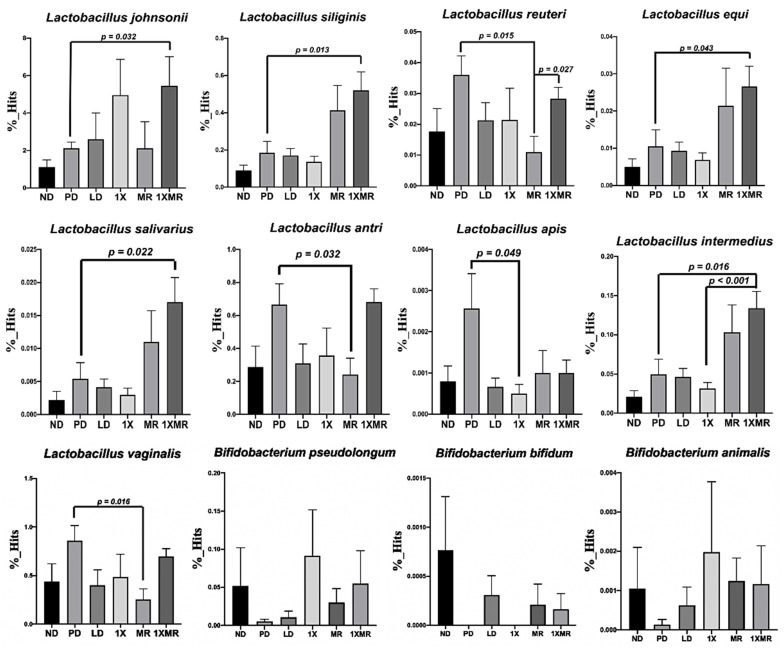
Comparison of specific bacteria identified in rats with 6-OHDA-induced lesions after 8 weeks of 1X, MR, and 1XMR treatment. Values are reported as mean ± SEM for 5 rats per group. ND, non-diseased; PD, untreated PD; LD, PD treated with 8 mg of L-DOPA; 1X, PD supplemented with the probiotic; MR, PD supplemented with MR as prebiotic; 1XMR, PD supplemented with a combination of 1X and MR.

Next, we further investigated the changes in specific species belonging to the altered genera detected by LEfSe analysis. The relative proportions of species belonging to the beneficial genera *Lactobacillus* and *Bifidobacterium* in the different groups are shown in Figure 6. Compared to the ND group, the PD group showed a tendency towards an increased abundance of *Lactobacillus* and lower abundance of *Bifidobacterium* species. Compared to the PD group, the 1X group showed a decreased abundance of *L. apis* (*p* = 0.049); the MR group showed a decreased abundance of *L. reuteri* (*p* = 0.015), *L. antri* (*p* = 0.032), and *L. vaginalis* (*p* = 0.016); and the 1XMR group showed an increased abundance of *L. johnsonii* (*p* = 0.032), *L. siliginis* (*p* = 0.013), *L. equi* (*p* = 0.043), *L. salivarius* (*p* = 0.022), and *L. intermedius* (*p* = 0.016). Although the changes were not significant (*p* > 0.05), the treated groups exhibited a tendency towards an increased abundance of beneficial Bifidobacterium species. Overall, these data show that probiotic supplementation (1X, RM, and 1XMR) reshaped the fecal microbiota composition of the rats with 6-OHDA-induced PD.

Spearman’s correlation analysis was performed to uncover potential correlations be-tween the bacterial taxa and the phenotypic PD parameters. A summary of the significant correlations between the parameters of PD progression and specific bacterial taxa at the genus level is shown in Table 2. The abundance of *Synergistes* correlated positively with endurance performance and catalase activity. Only *Eggerthella* was positively correlated with all the activity of all three antioxidant enzymes, SOD, GPx, and catalase. Various other genera, including *Bacteroides*, *Anaerostipes*, *Prevotella*, *Sutterella*, *Catenibacterium*, *Meganomonas*, and *Citrobacter,* were positively associated with only catalase activity. Only the *Lactobacillus* genus correlated negatively with catalase activity. However, at the species level, *Lactobacillus* showed varied correlations with the PD parameters, as summarized in Table 3. Specifically, *L. apis* and *L. reuteri* were positively correlated with contralateral rotation frequency and negatively correlated with endurance performance and SOD and GPx activity. Although the majority of *Lactobacillus* species showed negative correlation with the activity of the three antioxidant enzymes, the species *L. pentosus*, *L. japonicus*, and *L. plantarum* positively correlated with catalase activity.

Two *Bifidobacterium* species, *B. adolescentis* and *B. longum*, also positively correlated with catalase activity. These observations indicate that the beneficial or detrimental effects of *Lactobacillus* on the progression of PD could be species dependent. Taken together, these results further confirm that probiotic/prebiotic supplementation reshaped the fecal microbiota composition of the PD rats with 6-OHDA-induced gut dysbiosis. The alterations to the fecal microbiome due to supplementation may be related to the increased antioxidant activities observed in the 1X, MR, and 1XMR groups.

## 4. Discussion

Oxidative stress plays a major role in the progression of PD. An imbalance between ROS and antioxidant activity promotes inflammatory conditions in PD, which can create a vicious cycle and worsen neuronal cell death. Thus, treatments targeting the suppression of oxidative stress may delay or reduce the severity of PD. Probiotics are live microorganisms that exhibit antioxidant and anti-inflammatory activity. Additionally, the ability of probiotics to modify the gut microbiota may potentially help to impede the progression of PD, as recent evidence indicates a correlation between the progression of PD and gut dysbiosis [18]. In this study, we show that eight weeks of supplementation with the probiotic *Lactobacillus salivarius* AP-32 (1X), prebiotic medium residue (MR) obtained from AP-32 cell-free supernatant, or a cocktail containing both the probiotic and prebiotic (1XMR) alleviated the development of PD-associated motor deficits and dopaminergic neuronal loss (Figure 2) in rats with 6-OHDA-induced PD. These treatments increased serum antioxidant activity, lowered oxidative stress and inflammation, altered the fecal microbiota composition, and increased the concentrations of SCFAs in fecal samples. These results suggest that the probiotic and prebiotic supplements directly and indirectly suppressed oxidative stress by upregulating antioxidant pathways and by increasing antioxidant capacity, respectively. The direct pathway involves supplement-induced increases in the activity of host antioxidant enzymes and increased host production of antioxidant-related metabolites, specifically SCFAs. The indirect pathway involves a supplement-induced shift in the abundance of commensal bacterial species, which are associated with the production of microbial metabolites and the activity of antioxidant enzymes. This is the first study to link probiotic supplementation and a potential prebiotic to the optimization of antioxidant activity due to alterations in the fecal microbiota in a PD-like model.

Previous studies have shown that various probiotic strains can directly influence host antioxidant capacity via different mechanisms, such as chelation of metal ions [42], stimulation of the host antioxidant system, suppression of ROS-producing enzymes [43], modulation of antioxidant signaling pathways [44], and production of antioxidant metabolites [45,46,47]. Probiotics can improve the host antioxidant system by regulating lactic acid bacteria, such as *Lactobacillus*, which are known to inhibit oxidative processes by chelating Fe^2+^ or Cu^2+^ [42]. Additionally, the antioxidant enzymatic systems within probiotics could also play a role in reducing oxidative stress in the host. *Lactobacillus fermentum* E-3 and E-18 are reported to express Mn-SOD [43], which could decrease host mitochondrial ROS. Probiotics are also known to modulate antioxidant signaling pathways by stimulating phosphorylation of nuclear transcription factor-erythroid 2 related factor (NRF2) and hemeoxygenase-1 (HO-1). Activation of NRF2 and HO-1 leads to increased transcription of antioxidant enzymes [44] and thus enhances protection against oxidative stress. Moreover, probiotics can generate antioxidant metabolites, such as glutathione [45], folate [47], and butyrate [46]. In this study, our probiotic/prebiotic treatments directly increased the activity of host antioxidant enzymes, in accordance with previous studies [38,48,49]. Furthermore, Romo-Araiza et al. [49] reported that probiotic, prebiotic, and synbiotic supplementation could suppress the levels of the pro-inflammatory cytokines IL-1β and TNF-α. We observed supplementation with the probiotic, potential prebiotic, and their combination reduced the serum levels of TNF-α. Probiotic supplementation could decrease the level of nuclear factor kappa-light-chain-enhancer of activated B cells (NF-ĸB), which is the primary regulator of pro-inflammatory signaling [49]. Improved host antioxidant activity and reduced oxidative stress may also play roles in the suppression of TNF-α observed in this study. Additionally, the antioxidant and anti-inflammatory effects conferred by the probiotic and its residual medium may further prevent deleterious changes in mitochondrial function, as we described previously [38]. Overall, our results indicated that the probiotic, its medium residue, and the cocktail prevented the vicious cycle of mitochondrial dysfunction, oxidative stress, and inflammation in the rat model of PD, and these supplements eventually improved motor function and restored dopaminergic neurons.

The elevation in antioxidant activity due to the supplements could also possibly be due to alterations to the gut microbiota composition and modulation of SCFA production, as we observed that supplementation increased the total SCFA, butyric acid, and propionic acid concentrations. Probiotics can selectively inhibit the proliferation of harmful bacteria, which may suppress oxidative stress [50]. The production of SCFAs, such as butyrate and acetate, by the gut microbiota could inhibit excessive accumulation of ROS and nitric oxide. Inhibition of these free radicals may prevent mitochondrial dysfunction and the induction of cellular apoptosis [51]. Enhanced SCFA production could also suppress inflammation by regulating the expression of immune cell adhesion molecules, decreasing chemotaxis, and reducing cytokine release [52]. Moreover, alterations in the production of microbial metabolites induced by probiotic supplementation were previously speculated to activate the peroxisome proliferator-activated receptor gamma and elevate antioxidant and anti-inflammatory activity and in turn upregulate brain-derived neurotrophic factor, which promoted neuronal survival and proliferation [44].

In addition to the direct effects of the supplements on antioxidant capacity described above, indirect modes of action may also explain the neuroprotective effects observed following the 1X, MR, and 1XMR treatments. The indirect mechanism involves promotion of antioxidant and anti-inflammatory activity, which may be the result of treatment-induced changes in the gut microbiota composition. Our results showed that the probiotic/prebiotic treatments changed the fecal microbiota composition of the PD rats, and specific bacterial taxa positively correlated with host SOD, GPx, and catalase activity. The gut microbiota are known to protect the host from opportunistic pathogens via various processes, such as by occupying attachment sites, competitive exclusion, consumption of nutrient sources, and the production of antimicrobial substances [53]. Harmful bacteria proliferate excessively in the state of dysbiosis and induce the excessive release and circulation of endotoxins in the blood, which significantly elevate oxidative stress. The generation of ROS in response to microbial contact with gut epithelia is an extremely conserved phenomenon across phyla; this is the most common mechanism by which bacterial communities affect redox homeostasis in the host [54]. In humans and other animals, probiotics generally occupy the gastrointestinal tract [55,56], where they can modulate the composition of the microbiota and limit excessive proliferation of pathogenic bacteria, and these effects may possibly contribute to decrease oxidative stress. Furthermore, these changes may not only modify PD-associated dysbiosis but may also upregulate anti-inflammatory and antioxidant activity and contribute to neuroprotective effects in dopaminergic neurons. Gut microbiota dysbiosis is frequently reported in patients with PD, with increases in the relative abundance of *Lactobacillus* [57], *Bifidobacterium* [19], and *Akkermansia* [58] and decreases in the abundance of *Prevotellaceae* [22] and *Lachnospiraceae* [58] in fecal samples. Specifically, anti-inflammatory butyrate-producing bacterial genera, such as *Blautia*, *Coprococcus*, *Roseburia*, *Faecalibacterium*, *Blautia*, and *Ruminococcus* [18], are less abundant, while pro-inflammatory *Ralstonia* and *Clostridia* have been shown to be more abundant on the mucosa of patients with PD [58]. Thus, this state of gut dysbiosis could possibly be reversed with prolonged probiotic/prebiotic supplementation over several weeks.

Our study validated this hypothesis in rats with 6-OHDA-induced PD and showed that the probiotic, the potential prebiotic, and the prebiotic/probiotic cocktail decreased the proliferation of pathogenic bacteria, such as *Propionibacterium*, *Clostridium*, *Cylindriodes*, and *Ruminantium*, and increased the abundance of commensal genera, including *Ruminococcaceae*, *Bifidobacterium*, and *Faecalibacterium*. The changes in the composition of the microbiota after the three treatments were not identical. The MR treatment not only increased the abundance of commensal bacteria but also supported some pathogenic genera. The 1XMR treatment resulted in dramatic changes in which α- and β-diversities were reduced, and the relative abundance of multiple *Lactobacillus* species increased significantly. Therefore, 1X was the most effective treatment in terms of altering the fecal microbiota composition, as it specifically enriched the commensal *Ruminococcaceae* family and suppressed pathogenic genera while maintaining the abundance of *Lactobacillus* species.

*Lactobacillus* species are widely used as probiotics, and supplementation with these species for a few weeks can change the composition of the microbiota along the alimentary canal [33]. Hence, our study reasonably showed changes in the fecal microbiota composition of PD rats after eight weeks of supplementation with *L. salivarius* AP-32, either with or without the culture medium residue. Though other strains of *L. salivarius* have been used in clinical trials to treat metabolic syndromes, such as obesity and diabetes [59,60], neither *L. salivarius* nor prebiotics have been tested in clinical trials of patients with PD [61]. However, extending the findings of our pre-clinical study, enrichment of the *Ruminococcaceae* family and suppression of *Clostridium* by *L. salivarius* AP-32 with or without MR may be expected to reasonably reflect reversal of the gut dysbiosis associated with PD, at least to an extent.

Probiotic/prebiotic treatment-mediated changes in the gut microbiota composition may not only alleviate dysbiosis but could also increase anti-inflammatory activity by improving the intestinal integrity of the gut barrier and by preventing low-grade inflammation [62,63]. Enrichment of bacteria with SCFA metabolite-producing activity profoundly affected inflammatory responses via SCFA-G-protein-coupled receptor 43 interactions [64]. Moreover, enhanced production of SCFAs along with other metabolites, such as indole-3-propionic acid and ferulic acid, inhibited oxidative stress by balancing excessive generation of ROS [29]. We showed the 1X and 1XMR treatments enriched *Ruminococcaceae*, while the MR treatment enriched *Faecalibacterium*, *Mitsuokella*, *Bacteroides*, *Prevotella*, and *Bifidobacterium.* In fact, these commensal bacteria are known to produce SCFAs with anti-inflammatory properties [36]. For example, the abundance of *Faecalibacterium* from the *Ruminococcaceae* family was previously reported to be negatively correlated with the levels of inflammatory IL-6 and C-reactive protein [65,66,67]. The abundance of *Bacteroides* was associated with higher levels of C-reactive protein [68]. *Prevotella* are associated with improved glucose metabolism and SFCA production, mainly propionate [69,70]. *Bifidobacterium* was also associated with improved carbohydrate metabolism and lower levels of C-reactive protein [68,71]. Thus, enrichment of these specific taxa, which are involved in suppression of inflammation, strongly indicates that the fecal microbiota alterations due to supplementation may have subsequently led to upregulation of anti-inflammatory activity.

Changes in the composition of the gut microbiota might simultaneously upregulate antioxidant activity since probiotics are known to reduce oxidative stress by stimulating native antioxidant enzyme systems, including SOD, GPx, and catalase activity [50]. In the current study, we showed that *Eggerthella* was positively correlated with SOD, GPx, and catalase activity, while multiple other taxa, including *Prevotella* and *Meganomas,* were positively correlated with catalase activity in particular. *Eggerthella* are lignin-converting bacteria that have been reported to enhance SOD activity in rats [72] and exhibit catalase-inducing effects [73]. Interestingly, *Lactobacillus* correlated negatively with catalase activity. However, detailed analysis at the species level revealed that only specific *Lactobacillus* species were associated with the activity of all three antioxidant enzymes. For instance, *L. apis*, which was significantly reduced after 1X treatment, correlated negatively with GPx activity. Hence, the lower *L. apis* abundance induced by 1X may have upregulated GPx activity. The relative proportions of *L. reuteri* and *L. antri* species were significantly reduced by the MR treatment. These species correlated negatively with SOD and GPx activity; hence, suppression of these species by MR may have upregulated SOD and GPx activity.

Thus, the neuroprotective mechanisms may be related to increased production of SOD, GPx, and catalase by enriched and positively correlated taxa, which would increase the scavenging of hydroxyl radicals and hydrogen peroxide [50,74,75], while the butyrate from the enriched butyrate-producing taxa would inhibit histone deacetylases [76] and elevate peroxisome proliferator-activated receptor gamma-regulated Klotho expression [77], resulting in improvements in the metabolic profile and cellular oxidative status. Therefore, in addition to the direct effects imparted by the probiotic/prebiotic supplements, supplement-induced restructuring of the taxa may also contribute to increased anti-inflammatory and antioxidant activity, which in turn would decrease oxidative stress and provide neuroprotection in the rat model of PD.

It is important to emphasize that the gut dysbiosis in rodent models of 6-OHDA-induced PD still remains poorly characterized. Fecal samples from rats with 6-OHDA-induced PD tended to have an increased abundance of *Firmicutes* and decreased abundance of *Bacteroidetes* compared to normal rats though there was no significant difference in the *Firmicutes* to *Bacteroidetes* or the α-diversity and β-diversity of the microbiota. These findings are consistent with a recent study by Choi et al. [78], which reported no change in α-diversity or β-diversity but an increased abundance of *Bacteroides* and decreased abundance of *Lactobacillus* in 6-OHDA-lesioned rats. In contrast, we found that the relative abundance of *Bacteroides* was lower, and the relative abundance of *Lactobacillus* was increased in our PD rats.

Moreover, we observed lower abundances of *Faecalibacterium*, *Lachnospira*, *Phascolarc-tobacterium*, *Megamonas*, and *Paraprevotella* in the PD rats (data not shown). Some of these bacteria are associated with anti-inflammatory activity. For instance, *Faecalibacterium* and *Lachnospira* produce butyrate for energy production [18,66] and salicylic acid, which exerts anti-inflammatory effects [79]. *Phascolarctobacterium* is associated with lower concentrations of LPS-binding protein and C-reactive protein [80], and high circulating levels of LPS-binding protein indicate systemic inflammation [81]. In contrast, *Paraprevotella* and *Phascolarctobacterium* possess inflammatory activity. *Paraprevotella* produces succinic acid, which can induce inflammation through the production of interleukin-1β [82]. *Phascolarctobacterium* utilizes succinate produced by other bacteria and has been closely associated with *Paraprevotella* [82]. Overall, the abundance of both commensal and pathogenic bacteria are altered in 6-OHDA-induced gut dysbiosis.

It is essential to note that the gut and fecal microbiota composition present in rat models may not recapitulate the microbiota composition in human due to the differences of genetics, anatomy, physiology, diet, and activity between rats and humans; those differences may pose limitations. Thus, the observations in rat models may not be fully applicable to humans. On the other hand, the crosstalk between gut microbiota and the host can also be affected by the host dietary pattern, which makes it difficult to distinguish the interactions between the gut microbiota composition and the intervention given in specific conditions, such as diseases or supplementations. It is an advantage of using a rat model to eliminate the influences of diets on the gut microbiota composition, as the diet can be easily controlled in rat models. Hence, despite the limitations, rat models are still good proxies to study the dynamic of gut microbiota composition in specific condition.

## 5. Conclusions

Our data showed that *Lactobacillus salivarius* AP-32 supplementation suppressed oxidative stress and inflammation by directly increasing systemic antioxidant activity in rats with 6-OHDA-induced lesions. The probiotic supplementation also altered the fecal microbiota composition by enriching some commensals while suppressing some pathogenic bacteria. Among the commensal bacteria, a number of specific taxa positively correlated with SOD, GPx, and catalase activity, particularly *Eggerthella*, *Prevotella*, and *Meganomas.* Therefore, such changes in the fecal microbiota might indirectly increase antioxidant activity. Regardless of whether the mode of action is direct or indirect, increased antioxidant enzyme activity may prevent dopaminergic neuronal loss in the brain and subsequently alleviate PD motor deficits caused by 6-OHDA-induced lesions. Therefore, *Lactobacillus salivarius* AP-32 may represent a potential candidate or co-adjuvant for slowing the progression of PD. Our evidence also supported the recommendation of probiotic supplementation as a useful strategy to prevent the progression of PD through enhanced antioxidant activity. In light of these observations, this study indicated that probiotics and prebiotics may be clinically useful as treatments to delay the development of PD by reducing oxidative stress and inflammation.

## Figures and Tables

**Table 1:** Effects of 8 weeks’ supplementation with 1X, MR, and 1XMR on the serum levels of ROS and TNF-α and activity of antioxidant enzymes.

	ND	PD	LD	1X	MR	1XMR
ROS (% normal control)	100.0 ± 5.8	187.5 ± 11.0 ^a^	172.4 ± 19.0	105.0 ± 21.3 ^b^	113.7 ± 10.3 ^b^	107.4 ± 12.1 ^b^
TNF-α (pg/mL)	43.9 ± 1.5	64.9 ± 1.6 ^a^	59.9 ± 2.4 ^ab^	51.2 ± 1.7 ^ab^	53.7 ± 2.9 ^ab^	52.0 ± 2.3 ^ab^
SOD activity (mU/mL)	68.7 ± 8.3	43.7 ± 1.6 ^a^	47.0 ± 1.6 ^ab^	51.6 ± 2.8 ^ab^	49.8 ± 1.6 ^ab^	50.6 ± 1.1 ^ab^
GPx activity (mU/mL)	65.3 ± 4.4	53.9 ± 2.1 ^a^	58.5 ± 1.4 ^b^	60.5 ± 1.1 ^b^	58.6 ± 1.0 ^b^	59.3 ± 1.2 ^b^
Catalase activity (mU/mL)	4.8 ± 0.1	3.4 ± 0.3 ^a^	3.6 ± 0.5 ^a^	4.4 ± 0.6 ^b^	3.8 ± 0.3 ^a^	3.8 ± 0.1 ^ab^

Means ± SEM (*n* = 5) in rows and with difference superscript notations are significantly different (*p* < 0.05). ND, non-diseased; PD, untreated PD; LD, PD treated with 8 mg of L-DOPA; 1X, PD supplemented with the probiotic; MR, PD supplemented with MR as prebiotic; 1XMR, PD supplemented with a combination of 1X and MR; SOD, Superoxide dismutase; GPx, Glutathione peroxidase; TNF-α, Tumor necrosis factor-alpha; ROS, Reactive oxygen species.

**Table 2:** Correlations between the taxa abundance of bacterial genera and parameters of Parkinson’s disease progression.

Progression Parameters	Correlation	Bacterial Taxa
Time on the rod(s)	+	*Synergistes* (r = 0.353; *p* = 0.040)
SOD ^1^ activity (mU/mL)	+	*Eggerthella* (r = 0.497; *p* = 0.003)
GPx activity (mU/mL)	+	*Eggerthella* (r = 0.402; *p* = 0.018)
Catalase activity (mU/mL)	+	*Bacteroides* (r = 0.404; *p* = 0.017), *Anaerostipes* (r = 0.473; *p* = 0.004), *Prevotella* (r = 0.391; *p* = 0.022), *Sutterella* (r = 0.392; *p* = 0.021), *Catenibacterium* (r = 0.503; *p* = 0.002), *Megamonas* (r = 0.489; *p* = 0.003), *Citrobacter* (r = 0.517; *p* = 0.001), *Synergistes* (r = 0.450; *p* = 0.007), *Eggerthella* (r = 0.372; *p* = 0.030)
	−	*Lactobacillus* (r = −0.372; *p* = 0.030)

^1^ SOD, Superoxide dismutase; GPx, Glutathione peroxidase; +, positive correlations; −, negative correlations. Significant correlations were defined as *p* < 0.05.

**Table 3:** Correlations between the taxa abundance of bacterial species and parameters of Parkinson’s disease progression.

Progression Parameters	Correlation	Bacterial Taxa
Contralateral rotation (rotations/minute)	+	*Lactobacillus reuteri* (r = 0.343; *p* = 0.046), *Lactobacillus apis* (r = 0.380; *p* = 0.026)
Time on the rod(s)	−	*Lactobacillus apis* (r = −0.343; *p* = 0.046), *Lactobacillus amylolyticus* (r = −0.365; *p* = 0.033),
SOD ^1^ activity (mU/mL)	−	*Lactobacillus reuteri* (r = −0.383; *p* = 0.025), *Lactobacillus antri* (r = −0.393; *p* = 0.035), *Lactobacillus frumenti* (r = −0.343; *p* = 0.046), *Lactobacillus camelliae* (r = −0.385; *p* = 0.024), *Lactobacillus senmaizukei* (r = −0.366; *p* = 0.032), *Lactobacillus panis* (r = −0.378; *p* = 0.027), *Lactobacillus amylolyticus* (r = −0.421; *p* = 0.013)
GPx activity (mU/mL)	−	*Lactobacillus reuteri* (r = −0.360; *p* = 0.036), *Lactobacillus apis* (r = −0.351; *p* = 0.041), *Lactobacillus camelliae* (r = −0.376; *p* = 0.028)
Catalase activity (mU/mL)	+	*Bifidobacterium adolescentis* (r = 0.411; *p* = 0.015), *Bifidobacterium longum* (r = 0.341; *p* = 0.048), *Lactobacillus plantarum* (r = 0.483; *p* = 0.030), *Lactobacillus pentosus* (r = 0.395; *p* = 0.020), *Lactobacillus japonicus* (r = 0.381; *p* = 0.026)
	−	*Lactobacillus taiwanensis* (r = −0.371; *p* = 0.030), *Lactobacillus siliginis* (r = −0.387; *p* = 0.023), *Lactobacillus intermedius* (r = −0.429; *p* = 0.011), *Lactobacillus salivarius* (r = −0.399; *p* = 0.019), *Lactobacillus tucceti* (r = −0.400; *p* = 0.018), *Lactobacillus panis* (r = −0.362; *p* = 0.035)

^1^ SOD, Superoxide dismutase; GPx, Glutathione peroxidase; +, positive correlations; −, negative correlations. Significant correlations were defined as *p* < 0.05.

## Data Availability

Data are contained within the article.
